# Various topical drug combination assessed using a neurosensory stent for chronic intraoral neuropathic pain: a pilot study

**DOI:** 10.22514/jofph.2024.025

**Published:** 2024-09-12

**Authors:** Yair Sharav, Yuval Shalit, Rakefet Czerninski, Shirley Leibowiz, Doron J Aframian, Yaron Haviv

**Affiliations:** ^1^Department of Oral Medicine, Sedation and Imaging, Hadassah Medical Center, Faculty of Dental Medicine, Hebrew University of Jerusalem, 9112001 Jerusalem, Israel; ^2^Hebrew University-Hadassah School of Dental Medicine, 9112001 Jerusalem, Israel; ^3^Department of Pediatric Dentistry, Barzilai Medical Center, 7830604 Ashkelon, Israel

**Keywords:** Neuropathic pain, Topical treatment, Oral pain, Neurosensory stent

## Abstract

Chronic intraoral neuropathic pain (NP), often developing post-dental procedures, poses significant management challenges. The prevalent use of systemic treatments, with their frequent substantial side effects, emphasizes the need for alternative therapeutic strategies. Our aim is to explore the efficacy and adherence with a topical drug regimen delivered through a neurosensory stent (NS) for treating chronic neuropathic pain (NP) within the oral cavity. A retrospective analysis in addition to a telephone structured questionnaire conducted on patients with chronic intraoral NP treated at the Orofacial Pain Clinic, Hadassah Medical Center, between 2017 and 2020. A standard combination of lidocaine 2%, pregabalin 5%, ibuprofen 5% and optionally amitriptyline 2% was administered using a custom-made NS. Out of 12 participants, 6 reported more than 50% pain relief, indicating high effectiveness. Notably, females showed a more favorable response than males. 75% of patients used the NS consistently. No significant difference in pain relief was observed between the standard formula and the one with supplementary amitriptyline. The results highlight the potential of NS as an alternative, or adjunct treatment that may reduce the dosage of systemic medications for chronic NP. Additionally, the NS device can be used as an “escape drug”, or add-on, method if pain exacerbates under systemic therapy or if higher dose of systemic therapy causes serious side effects. Large scale prospective double-blind studies are required to substantiate the findings of this pilot study.

## 1. Introduction

Neuropathic Pain (NP) from damage or injury to the somatosensory system causes significant suffering, severe limitations to daily activities and reduces overall quality of life [1]. Specifically, in the trigeminal area we refer to pain associated with traumatic nerve injury as painful traumatic trigeminal neuropathy (PTTN). PTTN is a chronic neuropathic pain that may develop following injury to the trigeminal nerve, that result from dental, surgical or local anesthetic procedures or physical trauma, such as a motor vehicle accident. Following nerve injury, there are various mechanisms, including peripheral and central, as well as phenotypic changes and genetic predispositions that may contribute to the development of neuropathic pain [2]. Another condition with chronic pain of a neuropathic nature without any known cause is persistent idiopathic facial pain (PIFP) [3, 4]. NP affects 7–8% of the population, accounting for 20–25% of chronic pain cases [5]. NP is usually chronic in nature suited to long-term prophylactic treatment [6]. Antiepileptic medications like pregabalin and antidepressants such as duloxetine are commonly used systemically, and have significant side effects [6, 7]. Other options for pain control include medications such as lidocaine, anti-inflammatory drugs, α2 adrenergic receptor agonists and NMDA receptor agonists [4, 7]. Topical application is an attractive alternate administration route due to safety, high drug bioavailability at the application site, reduced side effects, ease of application and rapid onset of action [8]. Moreover, first pass metabolism within the gastrointestinal tract is avoided [9]. When comparing the systemic bioavailability of topical diclofenac sodium gel 1%, applied to the skin, to orally administered 50-mg tablets of diclofenac sodium in healthy volunteers, the systemic exposure from the gel was up to 17-fold lower than with tablets [10]. Furthermore, the reduced side effects enable long-term treatment and improve compliance and treatment success [11, 12]. Topical treatments for NP are currently regarded as second or third-line options, however, recent publications suggest these agents could be used more often as first-line treatments [9, 13]. Various studies have demonstrated the efficacy of topical treatments in reducing neuropathic pain [4, 14]. In some cases, a combination of therapeutic agents may be required for optimal results [14]. Local formulations may include agents commonly used orally or intravenously for neuropathic pain, such as lidocaine, antiepileptic drugs (*e.g.*, gabapentin), antidepressants (*e.g.*, amitriptyline), anti-inflammatory drugs, α2 adrenergic receptor agonists (*e.g.*, clonidine), and NMDA receptor agonists (*e.g.*, ketamine) [4, 14]. The topical agents target local peripheral tissues to exert a therapeutic effect on peripheral receptors. However, our understanding of the precise mechanisms and the intricate cellular and tissue-level processes underlying the localized (topical) action of drugs that are traditionally used in systemic pain management is notably insufficient. While we recognize the efficacy of these drugs when administered systemically, targeting pain at its central neural pathways, our grasp of how these same agents exert their effects locally within the oral mucosa and surrounding tissues remains elusive for most of them.

Topical drug application within the oral cavity entails specific consideration, due to salivary washout effect, mucus presence and mouth movements involved in swallowing, speaking and chewing, all represent challenges in trans-mucosal delivery [15]. Appropriate muco-adhesiveness is fundamental in the development of trans-mucosal drug-delivery systems, increasing drug contact and bioavailability at the treatment site [16].

One of the best solutions to all these intra oral limitations are custom-made splints, or “neurosensory stents” (NS) that confine the medication to the affected area, ensuring efficacy [14]. Even stents alone, with no other treatment may provide pain relief [17]. The optimal combination of therapeutic substances remains unknown, yet this treatment approach has many potential benefits. The data regarding topical intraoral treatment for NP is limited to expert opinions, case reports and one rat study [11, 18]. The following drugs have been used for intra-oral topical treatment: ketamine 4%, carbamazepine 4%, lidocaine 1%, ketoprofen 4%, gabapentin 4% and pregabalin 5–10% in a Lipoderm base. The majority of topical intraoral mixtures used clinically contain pregabalin (5%), lidocaine (or other anesthetics, *e.g.*, benzocaine) (2%) [19] and ibuprofen (5%) [20, 21]. Application is typically applied to the inside of the stent three to four times daily [11, 22]. This pilot study assesses the impact of local topical treatments on analgesic efficacy and adherence in patients with oral chronic NP, using an intraoral NS.

## 2. Material and methods

Patients were interviewed and examined at the Orofacial Pain Clinic, Faculty of Dentistry, Hadassah-The Hebrew University, Jerusalem, between 2017 and 2020. Most patients attending these tertiary clinics had been treated unsuccessfully in the community. Primary and resultant data were recorded on an intake form. Demographic data included gender, age and relevant medical status. Patients were asked to evaluate pain quality and intensity over the week preceding the appointment. Pain intensity was measured using a verbal pain scale (VPS), where 0 indicated no pain and 10 represented the worst pain imaginable. In order to assess pain quality, patients selected a descriptive term from the following list: electrical, stabbing, throbbing, or pressure routinely employed in our clinic [7, 8, 23]. Additional information regarding the location of the pain (mandible or maxilla), whether it was unilateral or bilateral, the onset of pain and its pattern (continuous or episodic) was documented.

### 2.1 Telephone questionnaire

A structured telephone questionnaire was used to systematically evaluate treatment outcomes. This included an assessment of treatment efficacy and patient compliance. High efficacy was considered as a reduction in pain of at least 50%, low efficacy was defined as a reduction of less than 50%. Long treatment adherence was specified as use of the device for more than a month, while adherence of less than a month was marked as short. The questionnaire asked for subjective descriptions of pain, intensity levels, duration of use and comparative effectiveness compared to previous treatments for the current pain condition. Additionally, patients were inquired about any particular side effects they might have experienced, using an open-ended question format.

### 2.2 Participants

The cohort included patients who received topical treatment for chronic neuropathic pain in the oral region via a neurosensory stent (NS). Patients were followed for at least three months with two clinic visits. Diagnoses were established by experienced oral medicine specialists with expertise in orofacial pain (YH and YaS). The potential participant pool was 15 patients, out of which 12 (80%) were available to follow-up and consented to participate in the study.

### 2.3 Local treatment

The treatment protocol involved a compounded formulation of 2% lidocaine, 5% pregabalin and 5% ibuprofen, with a modified formulation incorporating an additional amitriptyline HCl (Hydrochloric acid) 2%. An experienced pharmacist prepared the gel-like formulation, which was administered using a custom-fabricated NS (see Fig. 1). The stent was designed to ensure coverage of the affected tissue while accommodating an adequate amount of medication. Patients were instructed to apply a pea-sized amount of medication to the inner side of the stent daily, as needed, to address their pain.

**Fig. 1.** Custom-made splint or Neurosensory Stent (NS) for delivering topical therapeutics to alleviate neuropathic pain, targeting the mandibular gingiva. (A) on model; (B) in mouth [8].

### 2.4 Statistical methods

Descriptive and inferential statistics were performed according to the clinical diagnosis and the results of treatment over time. *T*-test for independent samples was conducted when the categorical independent variable only had 2 levels. Chi-square tests for independence were conducted to test the relationship between a categorical variable and a categorical variable/ordinal. Frequency distributions and percentages were used to describe the sample according to categorical demographic and clinical variables and ordinal variables. To examine the quantitative demographic and clinical variables, center (mean) and dispersion (standard deviation, minimum and maximum value) indices were used. The data analysis was done using SPSS version 28, produced by IBM, NY, USA.

## 3. Results

Of the 12 individuals in the cohort, 9 had chronic neuropathic pain attributed to trauma and defined as painful traumatic trigeminal neuropathy (PTTN) and 3 had persistent idiopathic facial pain (PIFP) of neuropathic nature [4, 24] (Table 1). All participants received topical treatment with custom-fitted splints, 8 with a formulation that included lidocaine 2%, pregabalin 5%, ibuprofen 5% and 4 had amitriptyline HCl 2% added to the above formulation. No difference in pain relief was found between the formulations (Table 2). There were 2 males and 10 females with a mean age of 51 ± 10.00 years. Pain onset was between 2 and 48 months, with an average of 12 ± 12.6 months. Pain severity, as measured by VPS, ranged from 5 to 10, with an average of 7.5 ± 2.0, as documented in Table 1.

**Table 1.** Characteristics of patients and pain based on subjective reports for each participant (N = 12).

Number	Age	Gender	Diagnosis	Pain onset (mon)	Pain quality	Attacks/continuous	Location	location-unilateral/bilateral	Primary VPS*	Pain waking from sleep	Regular mixture/modified mixture**	Additional systemic treatment***	Topical treatment adherence****	Subjective topical treatment efficacy
1	58	F	PTTN	6	Burning	Continuous	maxilla	unilateral	9	Yes	modified	Non	Long	high
2	42	F	PTTN	8	Burning	Continuous	mandible	unilateral	9	No	regular	AED	Long	Low
3	46	M	PTTN	2	Burning	Attacks	Both	unilateral	7	No	regular	Non	Short	No change
4	43	F	PIFP	6	Numbness	Attacks	maxilla	unilateral	5	Yes	regular	Non	Long	Low
5	72	M	PIFP	6	Burning	Continuous	mandible	unilateral	10	No	regular	AED	Short	Low
6	54	F	PTTN	6	Other	Attacks	Both	bilateral	7	No	modified	AED	Long	high
7	39	F	PTTN	6	Other	Attacks	mandible	unilateral	7	No	regular	None	Long	high
8	56	F	PTTN	12	Burning	Attacks	mandible	unilateral	5	No	regular	AED, SNRI	Long	high
9	40	F	PTTN	10	Stabbing	Continuous	mandible	unilateral	6	No	modified	SNRI	Long	high
10	60	F	PTTN	48	Burning	Continuous	maxilla	bilateral	10	No	regular	AED, SNRI	Long	high
11	57	F	PTTN	10	Burning	Continuous	maxilla	bilateral	5	No	regular	AED	Short	No change
12	46	F	PIFP	24	Burning	Continuous	mandible	unilateral	10	No	modified	AED, SNRI	Long	Low
Mean ± SD	51.0 ± 10	-	-	12.0 ± 12.6	-	-	-	-	7.5 ± 2.0	-	-	-	-	-

*Primary VPS—VPS (Verbal Pain Scale) at first meeting. **including amitriptyline HCl 2%. ***AED (Anti-Epileptic drug), SNRI (Serotonin-Norepinephrine Reuptake Inhibitor). ****Adherence-Long—more than 1 mount, Short-Up to 1 month. PTTN: painful traumatic trigeminal neuropathy; PIFP: persistent idiopathic facial pain; SD: Standard Deviation; F: Female; M: Male.

**Table 2.** Assessment of treatment efficacy based on demographic and pain characteristics.

Variable	Values	N (%)	High efficacy of topical treatment N (%)*	No change or low efficacy N (%)*	*p* value
Gender					
	Female	10 (83.3%)	6 (50%)	3 (25%)	0.046
	Male	2 (16.7%)	0 (0%)	3 (25%)	
Treatment adherence**					
	Short	3 (25%)	0 (0%)	3 (25%)	0.046
	Long	9 (75%)	6 (50%)	3 (25%)	
Age (mean ± SD)					
	Years	12 (100%)	51.17 ± 9.26	51.01 ± 11.59	0.489
Diagnosis					
	PTNP	9 (75%)	3 (42%)	4 (33%)	0.505
	PIFP	3 (25%)	1 (8%)	2 (17%)	
Quality of pain					
	Burning	8 (67%)	3 (25%)	5 (42%)	0.221
	Other	4 (33%)	3 (25%)	1 (8%)	
Pain pattern episodic/continuous					
	Continuous	7 (58%)	3 (25%)	4 (33%)	0.558
	Episodic	5 (42%)	3 (25%)	2 (17%)	
Location					
	Maxilla	4 (33%)	2 (17%)	2 (17%)	1.000
	Mandible	6 (50%)	3 (25%)	3 (25%)	
	Both	2 (17%)	1 (8%)	1 (8%)	
Laterality					
	Unilateral	9 (75%)	4 (33%)	5 (42%)	0.505
	Bilateral	3 (25%)	2 (17%)	1 (8%)	
Waking from sleep					
	Yes	2 (17%)	1 (8%)	1 (8%)	1.000
	No	10 (83%)	5 (42%)	5 (42%)	
Time of onset (mon)					
	≤6	6 (50%)	3 (25%)	3 (25%)	1.000
	>6	6 (50%)	3 (25%)	3 (25%)	
Formulation of Topical treatment***					
	Including Amitriptyline	4 (33%)	3 (25%)	1 (8%)	0.395
	Not including Amitriptyline	8 (67%)	3 (25%)	5 (42%)	
Primary VPS					
	≥7	4 (33%)	2 (17%)	2 (17%)	1.000
	<7	8 (67%)	4 (33%)	4 (33%)	
Long term efficacy (h)					
	While using the splint and sometime afterward (h)	4 (33%)	3 (25%)	1 (8%)	0.465
	Only while using the splint	3 (25%)	1 (8%)	2 (17%)	
	No improvement at all times	5 (42%)	2 (17%)	3 (25%)	
Additional systemic treatment					
	With systemic treatment (AED, SNRI)	8 (67%)	4 (33%)	4 (33%)	1.000
	Without systemic treatment	4 (33%)	2 (17%)	2 (17%)	

*“Treatment efficacy” determined using a questionnaire about the degree of effectiveness of the topical treatment. High efficacy a >50% pain reduction. **“Treatment adherence” determined using a questionnaire about usage, use for more than one month was considered “Long” and less than one month was considered “Short”. ***The topical formula included lidocaine 2%, pregabalin 5% and ibuprofen 5%, and an alternative combination also containing 2% amitriptyline. VPS: Verbal Pain Scale; PIFP: Persistent Idiopathic Facial Pain; SD: Standard Deviation; PTNP: Post Traumatic Neuropathic Pain; AED: Antiepileptic drugs Drug; SNRI: Serotonin-Norepinephrine Reuptake Inhibitors.

Two thirds [8] of the participants described their pain as predominantly burning and the remaining third [4] described their pain as pressing, throbbing, or stabbing. Continuous pain was experienced by 7 participants (58%), and episodic pain attacks were reported by 5 (42%). Pain localization varied: 4 participants (33%) identified the maxilla, 6 (50%) the mandible and 2 (17%) experienced pain in both jaws. Pain was unilateral in 9 participants (75%) and bilateral in 3 (25%). Half the study participants [6] reported high treatment effectiveness, and half [6] reported no change or low effectiveness. A significant relationship was found between gender and treatment efficacy (χ^2^ (1, 1) = 4.001, c = 0.577, *p* = 0.046); the treatment was more effective in females. Long treatment adherence (more than a month) was reported by 75% [9] participants, while 3 (25%) reported short adherence (less than a month). A significant relationship was found between duration of splint use and treatment efficacy, (χ^2^ (1, 1) = 4.001, c = 0.577, *p* = 0.046), those with longer use experienced greater efficacy. Compared to previous treatments, 6 participants (50%) reported better effectiveness, 4 (33%) reported no difference, and 2 (17%) reported lower effectiveness. In terms of pain relief time, 4 participants (33%) experienced pain relief while using the product and for some hours after removing the NS, 3 (25%) experienced pain relief only when using the NS, and 5 (42%) experienced no improvement at all. Sleep disturbance due to pain was reported by 2 participants (17%), while the majority (83%) did not experience nocturnal awakenings (Table 2). A chi-square test for non-dependence was conducted and a clear relationship was found between waking up from sleep due to pain and whether the drug helped over time, (χ^2^ (1, 1) = 4.800, c = 0.632, *p* = 0.028). Those waking less frequently had more relief from the NS. The use of concurrent systemic treatment, in 8 out 12 participants, affected treatment outcomes. A significant relationship was found between gender and the efficacy of the treatment in relation to additional systemic medications (χ^2^ (1, 2) = 8.001, c = 0.816, *p* = 0.018), *i.e.*, efficacy was better for females also using systemic medications.

## 4. Discussion

This pilot study examined the effect of local topical medications on painful traumatic trigeminal neuropathy (PTTN) or persistent idiopathic facial pain (PIFP) neuropathic in nature, in the oral cavity. Individually tailored neurosensory stents (NS) is one of the best ways for maintaining topical medication in the preferred location within the oral cavity; considering the unique environment of the mouth. Their use enhances the duration of contact between the medication and the mucosal surface, simultaneously serving as a barrier against potential irritants [22]. Moreover, such appliances are designed to limit the spread of the delivered medications by the continuous flow of saliva in the oral cavity allowing precise application. Furthermore, the stents promote the utility of creating bespoke combinations of approved drugs for topical use. The thin device configuration ensures patient comfort while providing effective protection and therapeutic benefits [20, 21, 25]. Furthermore, titration for achieving effective levels is not required. Additionally, topical medications usually do not exhibit significant drug interactions and are associated with negligible side effects, barring occasional local allergies or rashes [9, 10, 26, 27, 28]. This facilitates long-term treatment, enhancing patient adherence and, consequently, the overall success of the treatment [11].

The potential effect of combining multiple therapeutic agents, typically utilized in the management of systemic neuropathic pain (NP) in a topical formulation was examined. An ointment with a mixture of lidocaine, ibuprofen, pregabalin and in some instances with amitriptyline was formulated for patients with treatment-resistant chronic oral NP. The inclusion of topical amitriptyline in the ointment did not yield a statistically significant increase in therapeutic efficacy. Half the participants (6 out of 12) reported high effectiveness, and had more than a 50% drop in pain scores. Additionally, 50% of the participants indicated that this treatment was more effective than previously used systemic drugs such as AEDs and SNRIs. Most of the patients (8 out of 12) used the topical treatment in addition to ongoing systemic treatment for pain. The majority of patients experienced pain relief while applying the topical ointment in conjunction with the splint, and this only persisted for a short duration after splint removal. This may imply that the NS device can be used an “escape drug”, or add-on, method if pain exacerbates under systemic therapy or if higher dose of systemic therapy causes serious side effects. In this small cohort, primarily comprising females, the female participants exhibited a more favorable response to the treatment. This observation is in concordance with previous studies showing the influence of gender on the efficacy of topical treatments, such as capsaicin patches for HIV-associated neuropathy. Indeed, evidence indicates that gender is a significant predictor of analgesic response, underlining the importance of considering gender differences in pain management Strategies [29].

Notably, in 50% of patients the treatment continued for over month. However, it remains unclear whether the improvement in pain levels was a motivating factor for continued treatment adherence or if early discontinuation of treatment contributed to a reduction in therapeutic benefits. Patients reporting mild to major pain reduction had higher adherence rates than those with complete pain relief or none at all [30]. Local treatments for NP probably lead to greater patient compliance than orally administered drugs due to fewer side effects. It may lead to minor and rare side effects such as mild irritation, infrequent allergic reactions, occasional taste alterations, and rare instances of dry mouth. More severe side effects, like oral mucositis and significant systemic absorption, are extremely rare. In this study, none of the patients reported any adverse effects.

There are evidences suggesting lay populations believe that application of treatments directly to the painful site significantly improves pain relief, probably leading to an increase in the placebo effect [22, 31]. The inclusion of topical amitriptyline in the ointment did not yield a statistically significant increase in therapeutic efficacy. Additionally, the study did not identify any specific pain-related or patient characteristics that were predictive of treatment success, as detailed in Table 2. However, the small cohort size means these findings are preliminary, and definitive conclusions cannot be drawn. Indeed, lidocaine HCl is a relatively safe and effective anesthetic and frequently used in local oral-mucosal pain [32]. Nevertheless, the precise mechanism by which the topical regimen alleviates pain is not fully understood, it is hypothesized that lidocaine may numb the pain receptors in the oral cavity, thereby reducing pain sensation [13]. Concurrently, ibuprofen potentially reduces inflammation at the affected site, contributing to pain relief. Regarding pregabalin, despite its frequent mention alongside its related compound, gabapentin, in numerous studies as a key topical intraoral medication for neuropathic pain [22, 28], the specifics of its mechanism of action at the peripheral level, distinct from its effects on the central nervous system (CNS), are yet to be elucidated.

The study’s limitations stem from its small sample size and the majority of female participants. This composition restricts the ability to generalize results and makes gender-based comparisons inconclusive. There are no control groups such as NP patients using a splint without medication, or only using the Lipoderm base. These considerations may contribute to the understanding of the effect of the medication in comparison to the mechanical effects of NS.

## 5. Conclusions

In conclusion, topical application of a compound containing lidocaine 2%, pregabalin 5% and ibuprofen 5%, with or without the addition of amitriptyline 2% HCl, within a neurosensory stent, demonstrates promising effectiveness in managing intraoral neuropathic pain, particularly in women. A significant aspect of this research is the possible utilization of the NS device as an adjunct “escape drug” in tandem with systemic medications for neuropathic pain.
